# Widespread Reassortment Shapes the Evolution and Epidemiology of Bluetongue Virus following European Invasion

**DOI:** 10.1371/journal.ppat.1005056

**Published:** 2015-08-07

**Authors:** Kyriaki Nomikou, Joseph Hughes, Rachael Wash, Paul Kellam, Emmanuel Breard, Stéphan Zientara, Massimo Palmarini, Roman Biek, Peter Mertens

**Affiliations:** 1 Vector-Borne Viral Diseases Programme, The Pirbright Institute, Pirbright, Woking, United Kingdom; 2 MRC–University of Glasgow Centre for Virus Research, Glasgow, United Kingdom; 3 Wellcome Trust Sanger Institute, Wellcome Trust Genome Campus, Hinxton, Cambridge, United Kingdom; 4 Division of Infection and Immunity, Research Department of Infection, University College London, London, United Kingdom; 5 French Agency for Food, Environment and Occupational Health and Safety (ANSES), Maisons-Alfort, France; 6 Institute of Biodiversity, Animal Health and Comparative Medicine, Boyd Orr Centre for Population and Ecosystem Health, College of Medical, Veterinary and Life Sciences, University of Glasgow, Glasgow, United Kingdom; Cincinnati Children’s Hospital Medical Center, UNITED STATES

## Abstract

Genetic exchange by a process of genome-segment ‘reassortment’ represents an important mechanism for evolutionary change in all viruses with segmented genomes, yet in many cases a detailed understanding of its frequency and biological consequences is lacking. We provide a comprehensive assessment of reassortment in bluetongue virus (BTV), a globally important insect-borne pathogen of livestock, during recent outbreaks in Europe. Full-genome sequences were generated and analysed for over 150 isolates belonging to the different BTV serotypes that have emerged in the region over the last 5 decades. Based on this novel dataset we confirm that reassortment is a frequent process that plays an important and on-going role in evolution of the virus. We found evidence for reassortment in all ten segments without a significant bias towards any particular segment. However, we observed biases in the relative frequency at which particular segments were associated with each other during reassortment. This points to selective constraints possibly caused by functional relationships between individual proteins or genome segments and genome-wide epistatic interactions. Sites under positive selection were more likely to undergo amino acid changes in newly reassorted viruses, providing additional evidence for adaptive dynamics as a consequence of reassortment. We show that the live attenuated vaccines recently used in Europe have repeatedly reassorted with field strains, contributing to their genotypic, and potentially phenotypic, variability. The high degree of plasticity seen in the BTV genome in terms of segment origin suggests that current classification schemes that are based primarily on serotype, which is determined by only a single genome segment, are inadequate. Our work highlights the need for a better understanding of the mechanisms and epidemiological consequences of reassortment in BTV, as well as other segmented RNA viruses.

## Author Summary

Segmented viruses have genomes that are separated into multiple segments, comparable to chromosomes in higher organisms. When two segmented viruses of the same species infect the same cell, their progeny may incorporate segments picked up from the “parental” viruses. This process is called “reassortment” and represents an important way for segmented viruses to evolve. Whereas reassortment has received a lot of attention in certain segmented viruses, especially influenza A, its frequency and biological consequences remain poorly understood for most of the others. Here, we present a comprehensive analysis of the reassortment patterns in bluetongue virus, an important pathogen of livestock, during its repeated emergence in Europe in recent decades. We confirm earlier reports that reassortment is common and can involve segments derived from live vaccines used to control outbreaks. However, the mixing of viral genomes is not strictly random and reassortment is commonly followed by novel adaptive changes in the progeny virus. This points to important functional links (paired associations) between certain segments. Our findings have important implications for the classification and control of segmented viruses and generate new insights and hypotheses about the biological interactions among different parts of the bluetongue virus genome.

## Introduction

Reassortment is an important evolutionary process in segmented RNA viruses that can occur when two viruses (of the same species) co-infect a single host cell [1]. This provides an opportunity for their genome segments to be exchanged and packaged together into progeny viruses that are therefore genetically distinct from their parental virus strains. By combining potentially divergent genetic material, reassortment can quickly generate novel virus phenotypes, with potentially dramatic biological consequences, including an altered ability for immune escape, changes in host or vector range, changes in transmissibility and altered virulence or pathogenicity [2–6]. Although some taxa, such as influenza viruses, have received considerable attention in this respect, our understanding of reassortment, including its natural rate, evolutionary and epidemiological consequences, still remains relatively poor for most segmented viruses [1].

Members of the genus *Orbivirus*, within the family *Reoviridae*, have ten genome segments. *Bluetongue virus* is the prototype species of the genus and includes several viruses which are the causative agents of bluetongue (BT), a major disease of wild and domestic ruminants [7,8]. BTV is an arthropod-borne virus spread between its mammalian hosts primarily by competent species of biting midges (*Culicoides* spp.). It can sometimes also be transmitted *via* an oral route, or vertically in sheep and cattle, and some serotypes may be transmitted horizontally by direct contact [9].

The ten linear segments of double-stranded RNA (dsRNA) that comprise the BTV genome are identified as genome-segment 1 to genome-segment 10 (Seg-1 to Seg-10) in order of decreasing size. Collectively the BTV genome segments encode seven structural (VP1-VP7) and four non-structural proteins (NS1-NS4) that are expressed during virus replication in vertebrate or insect cells [10,11]. The highly variable outer-capsid protein VP2 (encoded by Seg-2) is of particular relevance because it determines the virus-serotype, which is important for selection of appropriate vaccines and represents an important component of current strain identification for BTV [12–15]. To date, 27 distinct serotypes have been characterised. In addition, BTV are further distinguished into different ‘topotypes’, including the major “eastern” (e) and “western” (w) groups, as well as several additional groups and sub-groups [16].

There is a considerable body of prior work, mainly from North America and Australia, demonstrating that BTV reassortment occurs in the field and that it can take place in the *Culicoides* vector as well as the ruminant host [17–20]. The earliest of these studies were not based on sequencing [21–25] and thus could not provide exhaustive information about the segments involved. Subsequent work based on sequence data confirmed earlier findings but focussed on specific segments and often involved partial sequences [26–29]. More recently, sequence data for all ten genome segments for representative isolates of the ten BTV serotypes isolated in Australia produced evidence of frequent reassortment at the genomic scale [30,31] based on a relatively small number of isolates (<30). While collectively, these data highlight the global importance of BTV reassortment [32], the lack of appropriately large genomic data sets has so far precluded a quantitative assessment of reassortment in terms of its frequency and evolutionary implications.

Our recent work involving European strains of BTV-1 and BTV-8 (both western topotype) has demonstrated that all segments can reassort in infected tissue cultures and that these strains show few constraints limiting or preventing particular reassortment combinations from arising and being viable [15]. However, we found evidence that certain reassortants occur more frequently than expected by chance, suggesting that these combinations are either favoured by selection or because some genome-segments are more likely to be packaged together. Overall, these *in vitro* studies raise the question of how conditions *in vivo*, where additional constraints are imposed by antiviral immune responses and need for the virus to replicate in both the vertebrate host and the arthropod vector, restrict the diversity of reassortants arising and circulating in the field.

Bluetongue virus has been documented on every continent except Antarctica. Before 1998, BTV outbreaks in Europe tended to be localised, caused by a single serotype and were usually limited to a few years duration. Examples include outbreaks in Cyprus (1943; BTV-3 and BTV-4), Turkey (1944–1947; BTV-4), the Iberian Peninsula (1956–1960; BTV-10) and Greece (1979–1980; BTV-4)[33]. However, since 1998, Europe has experienced multiple incursions caused by different BTV serotypes, as well as different topotypes and lineages within individual serotypes. This expansion in the distribution of BTV is thought to reflect a range of environmental factors, including changes in climate, range expansion of *Culicoides* vector species, global transport networks, and increased trade and travel [34,35].

Bluetongue incursions into Europe have occurred *via* several distinct routes: strains of BTV-1 (eastern topotype: e), BTV-4 (western topotype: w), BTV-9(e) and BTV-16(e) have all invaded the eastern Mediterranean, possibly *via* Turkey. Other strains of BTV-1(w), BTV-2(w), BTV-4(w) and BTV-9(w) have entered western Europe from northern Africa, either *via* Sicily, Italy and the western Mediterranean islands or into the Iberian peninsula from Morocco. In 2006, a strain of BTV-8(w) [NET2006/01], (that was related to BTV-8 from Nigeria [NIG1992/07]) was detected in the Netherlands. This represented the start of the first recorded BT outbreak in Northern Europe and indicated a route of entry to the region directly from sub-Saharan Africa that did not involve step-wise progression through southern Europe or the Mediterranean region. Finally, multiple live vaccine strains (including BTV-2w, BTV-4w, BTV-9w, BTV-16e) were also used to combat disease outbreaks in southern Europe, resulting in local transmission and spread of these viruses. Subsequently BTV-6(w), BTV-11(w) and BTV14(w) which are all closely related to BTV vaccine strains, were also detected in northern Europe, although their route of entry is unclear. In addition with the development of better detection and identification methods, two novel serotypes of BTV have been identified in Europe (BTV-25 and BTV-27), although it is unclear if they represent new introductions, or already existed in the region for long periods [36,37]. Information based on partial genome sequences from a limited numbers of BTV field strains, indicated that reassortment between some strains had occurred (e.g. for BTV-16(e) in Italy 2002 or BTV-1(w) and BTV-4(w) in Sardinia 2012 [38–40]).

Modified live-attenuated vaccines (MLVs) were only used in a few of the European countries that experienced BTV outbreaks since the 1940s. This includes Italy, where vaccines against BTV-2, 4, 9 and 16 were deployed, France (Corsica) where vaccines against BTV-2, 4 and 16 were used, and Spain and Portugal where vaccines against BTV-4 were used [41]. These MLVs were derived from strains isolated in South Africa, Pakistan or Australia. BTV strains that are closely related to MLVs have been shown to spread in the field, including BTV-2 [42], BTV-6 [43], BTV-11 [44] and BTV-16 [45]. In addition, MLVs are commonly used in North African and Middle Eastern countries, many of which report regular outbreaks caused by a variety of different BTV serotypes.

In this study, we have investigated the role of reassortment in BTV evolution following the virus’ multiple recent incursions into Europe. While Europe was considered to be free from Bluetongue from 1980 to 1998, multiple BTV strains have co-circulated since 1998, all of which were characterised soon after introduction. Combined with the use of MLVs, some of which have also been transmitted in the field, this has generated a unique research opportunity to study BTV reassortment *in vivo* and to examine its effect on the evolutionary and epidemiological dynamics of BTV. We have conducted sequence analyses of >150 full BTV genomes, including multiple European field samples collected as far back as the 1950’s. This has allowed us to i) estimate the frequency of reassortment following recent European colonisations; ii) test for non-random associations of specific genome segments during reassortment; iii) examine whether reassortment triggers selectional responses in the BTV genome; iv) determine the involvement and role of MLVs in reassortment.

## Results

### Frequency of reassortment among European serotypes

This study uses full genome sequences that we have generated from 116 BTV isolates from Europe and the Mediterranean region and Africa countries (collected during 1958–2012) as well as 4 monovalent BTV vaccine strains, in addition to sequences of 26 BTV reference strains and other isolates and vaccines available in GenBank at the time of this study. In total, consensus sequences for individual genome segments of 178 BTV isolates for Seg-2 and Seg-6 and from 163 BTV isolates for all other segments were included in our analyses. Details of the date and location of sample collection, host species, and passage history are shown in S1 Table along with GenBank Accession numbers.

**Fig 1:**
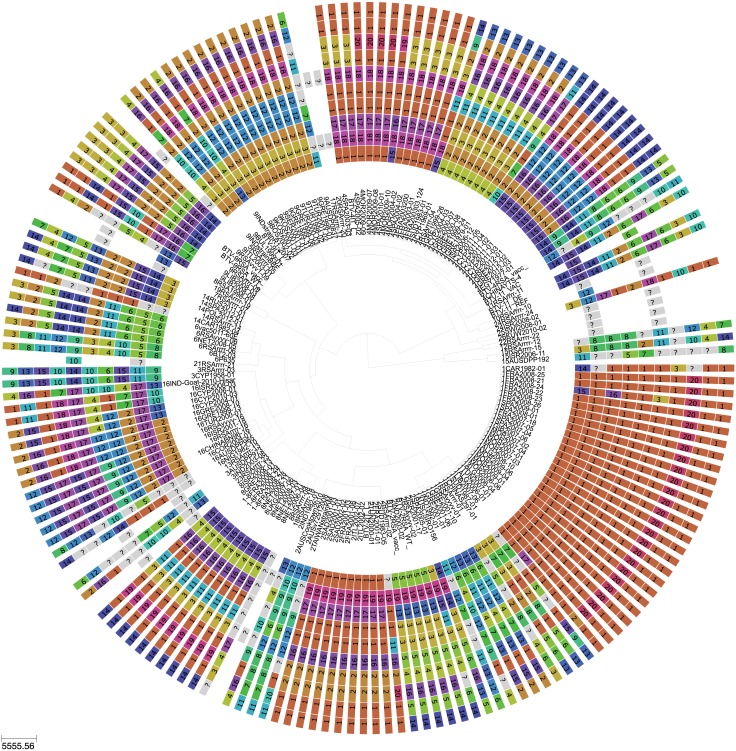
Phylogeny of BTV Seg-2 and inferred genomic reassortment patterns. Bayesian maximum clade credibility tree of 118 viruses from Europe and the Mediterranean region as well as 60 global viruses included for comparison. Cluster assignment of each segment based on phylogenetic lineages identified using Cluster Picker [46]. Sequences of the same segment that were assigned to the same cluster are indicated by shared colours and numbers (with numbers being arbitrary and not corresponding to serotype). There is no significance to sequences from different segments sharing the same colour or number. For some reference strains, only data for Seg-2 and Seg-6 were available.

**Fig 2:**
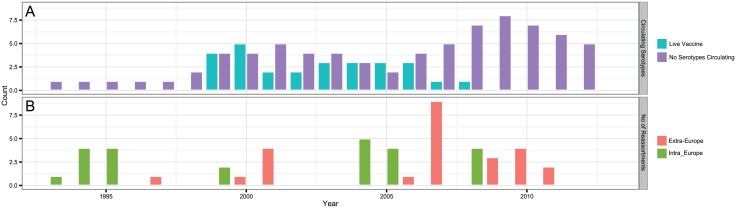
BTV strains and reassortment events in Europe since 1993. (A) Number of known strains circulating in Europe and number of known live-attenuated vaccines used in Europe since 1993; (B) Inferred number of reassortments per year occurring inside (Intra-Europe) and outside Europe (Extra-Europe) every year in lineages assigned as European based on molecular clock calibration of BTV phylogenies. The date provided for a reassortment event corresponds to an upper bound.

Based on the most parsimonious reconstruction of segments onto Seg-2, it is clear that reassortment among BTV strains is a common event, resulting in highly admixed genomes (Fig 1). Using molecular clock calibrations, we find that the majority of reassortment events detected in our data set occurred after the mid-1990’s, coinciding with emergence of new strains and serotypes in Europe, as well as with the use of different live vaccine strains that have been applied, or have been circulating in the region (Fig 2). Up until 1998, BTV-4 was the only serotype known to be present in Europe over multiple years (Cyprus 1964–2011), although BTV-3 was previously detected in 1958, and BTV-10 caused a single outbreak in the Iberian peninsula between 1956 and 1960. According to the estimated dates associated with the corresponding tree nodes, nine reassortants emerged between 1993 and 1998 so prior to serotypes other than BTV-4 becoming established in Europe. This suggests that additional strains may have been already present during that period but escaped detection. In light of these results, we use 1993 as our lower temporal cut-off point for quantifying reassortment in Europe. Since 1993, a total of 49 reassortment events were detected in European lineages, providing an average rate of 0.05 per genome per year. This compares to an estimated overall evolutionary rate of 3.84 substitutions per genome per year (S2 Table) that is broadly consistent with rates previously reported for BTV [31, 32].

For nine highly passaged viruses analysed (>40 passages), the original isolation dates were within the confidence limits of the ‘estimated dates’ for most samples. Based on all isolates, the number of passages did not significantly correlate with the difference in time between the estimate and the actual date of isolation, suggesting that passage in cell cultures made a negligible contribution to their molecular evolution.

### Frequency and non-random associations among BTV genome segments

**Fig 3:**
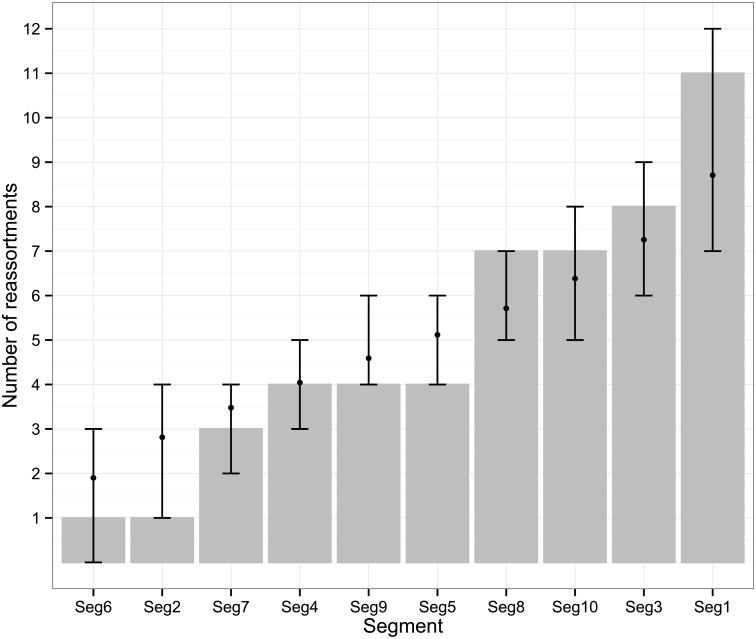
Number of inferred reassortment events per BTV segment in Europe since 1955. All frequencies estimated relative to Seg-2; for Seg-2 the phylogeny of Seg-6 was used as the reference point instead. Bars represent expected values based on 10,000 simulations.

**Fig 4:**
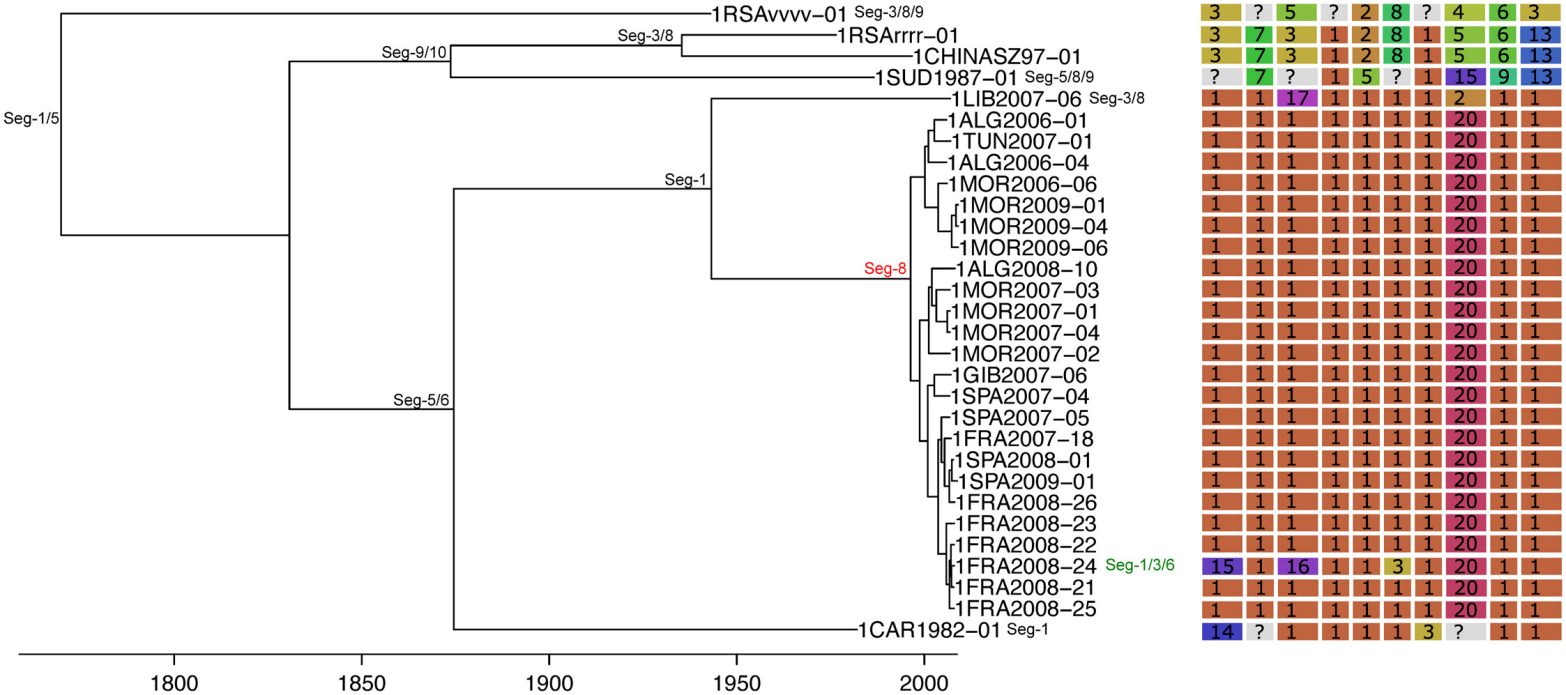
Time-scaled Seg-2 phylogeny of European BTV-1(w) isolates. Shown is the maximum clade credibility tree estimated in program BEAST with branch lengths scaled in years. Inferred reassortment events (based on changes in genetic cluster assignment for one or more segments, shown on the right) are indicated by depicting the reassorting segment next to the respective tree node, with colours indicating whether events were inferred to have occurred within Europe (green) or outside Europe (red), both from 1993 onwards. Events in black indicate events prior to 1993 for which date and location are uncertain. Question marks in the genetic cluster assignments represented cases where the assignment was ambiguous (see Methods for further details).

**Fig 5:**
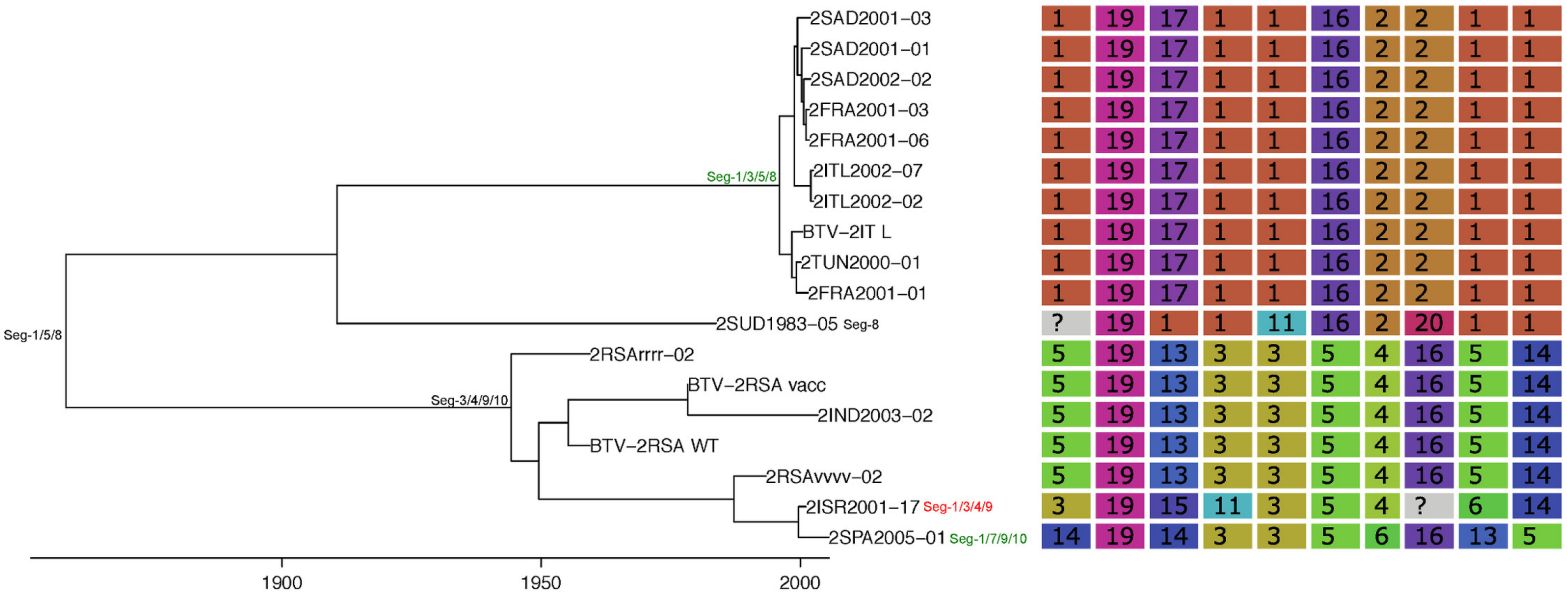
Time-scaled Seg-2 phylogeny of European BTV-2(w) isolates. See Fig 4 for details.

**Fig 6:**
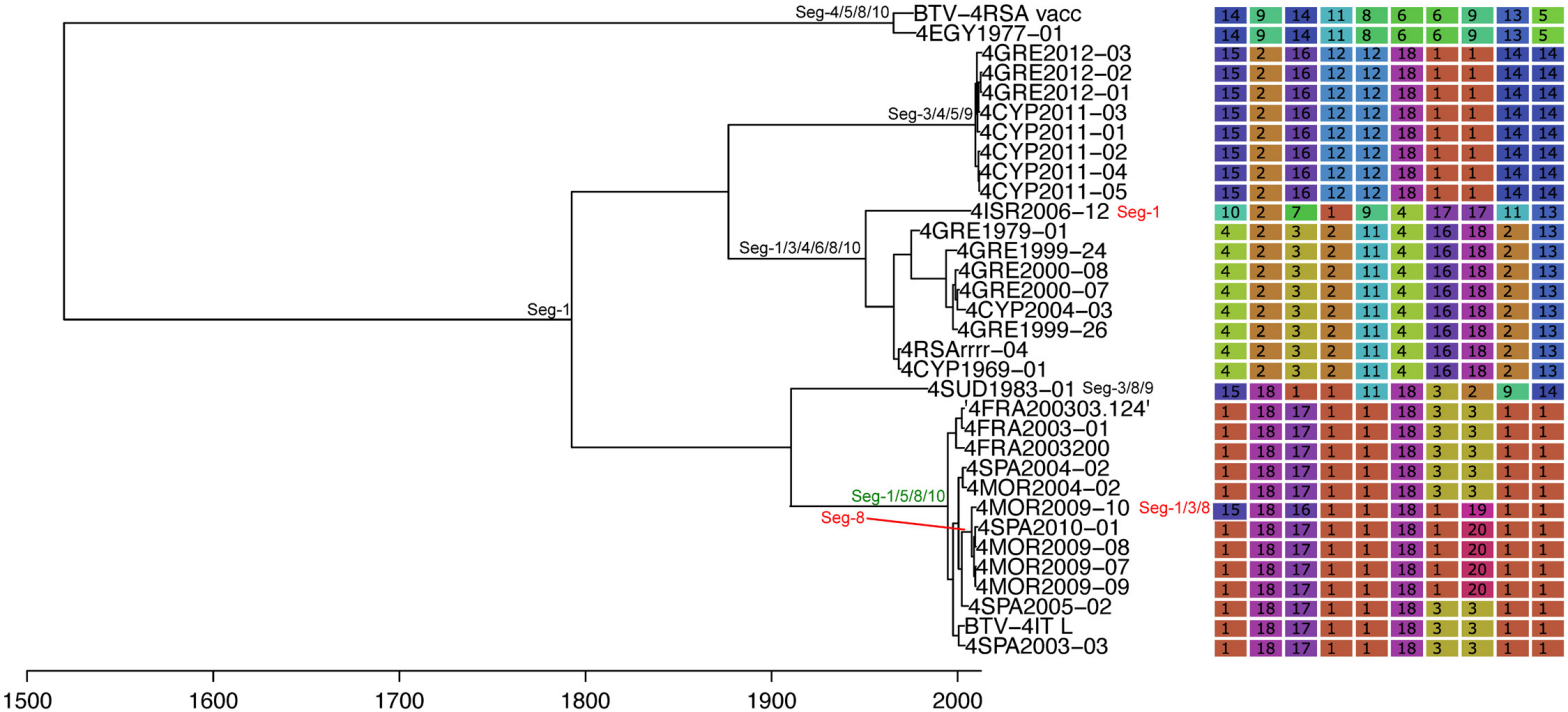
Time-scaled Seg-2 phylogeny of European BTV-4 isolates. See Fig 4 for details.

**Fig 7:**
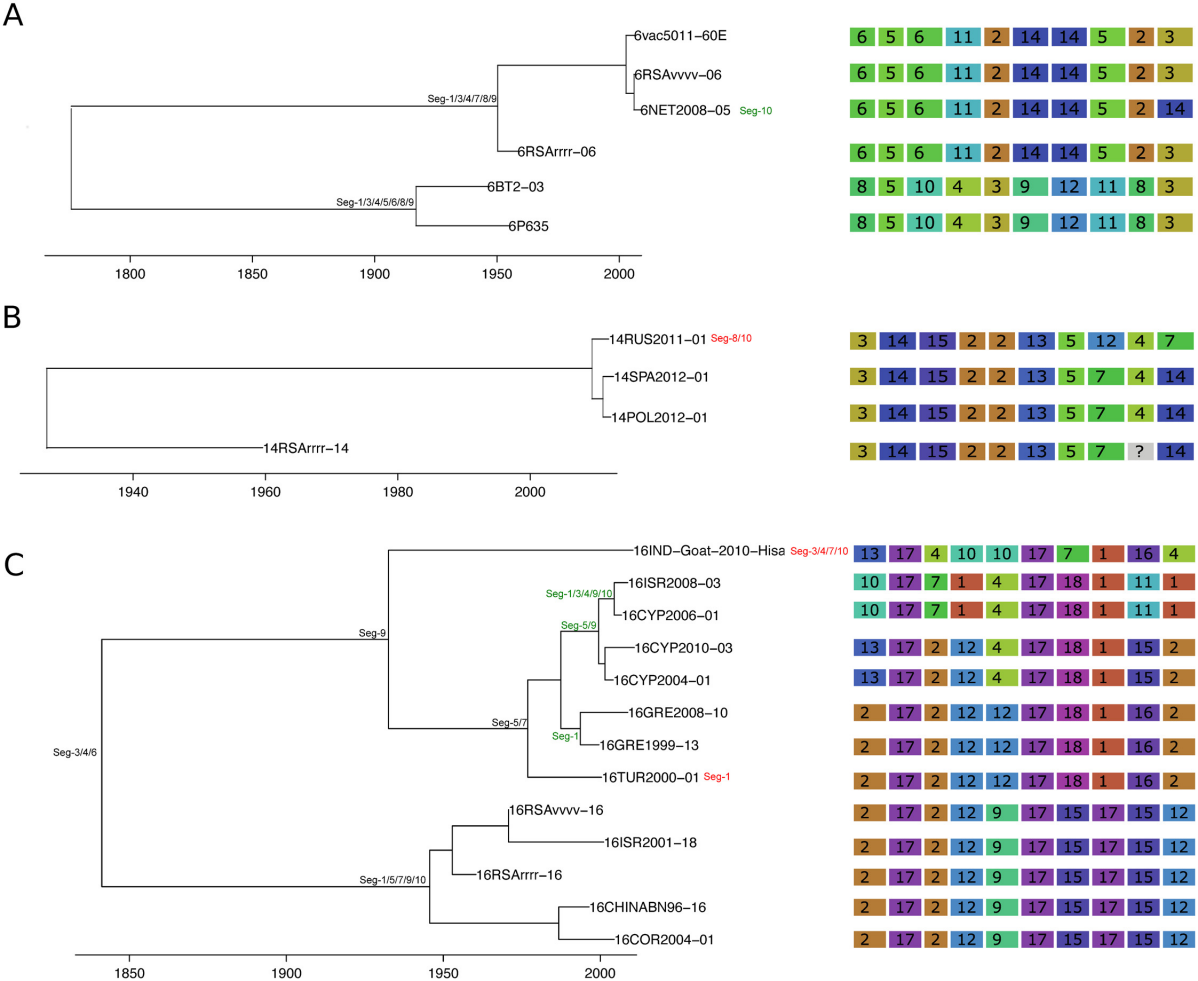
Time-scaled Seg-2 phylogenies of European isolates of BTV-6 (A), BTV-14 (B), and BTV-16 (C). See Fig 4 for details.

The frequency at which segments were involved in reassortment was variable, ranging from a single instance (Seg-2 and Seg-6) up to eleven (Seg-1). However, observed frequencies largely fell within the 95% CI of the simulated data, indicating no particular bias in terms of some segments reassorting more or less frequently than expected (Fig 3). The number of genome-segments reassorting around the same time also varied. In most cases, a single segment reassorted, but up to seven segments were exchanged at the same node in a European lineage of BTV-6 relative to Seg-2 (Figs 4–7), S1 Fig). For five serotypes or topotypes, no reassortment events were detectable within Europe (S2–S6 Figs).

Taking variation in reassortment frequency into account, genome segments were found to be reassorting non-randomly overall (observed C = 0.36955, expected C = 0.50718, p = 0.0125). However, none of the individual segment pairs showed significant associations after controlling the false discovery rate (Table 1).

**Table 1:** Negative and positive pairwise associations among BTV segments during reassortment. None of the p-values were significant after correcting for multiple testing using the false discovery rate method. Reassortment was inferred relative to Seg-2, except for associations involving Seg-2, which were inferred relative to Seg-6. Associations between Seg-2 and Seg-6 are therefore not defined.

	Seg-1	Seg-2	Seg-3	Seg-4	Seg-5	Seg-6	Seg-7	Seg-8	Seg-9	Seg-10
Seg-1		-0.25	0.25	0.15	0.15	0.19	0.05	-0.3	0.15	-0.06
Seg-2			-0.22	-0.18	0.31	NA	-0.12	0.35	-0.18	0.31
Seg-3				0.6	0.06	0.27	0.2	-0.03	0.06	-0.03
Seg-4					0.04	-0.13	0.48	-0.43	0.36	0.4
Seg-5						-0.13	0.12	0.12	0.04	0.12
Seg-6							-0.11	-0.19	-0.13	-0.19
Seg-7								-0.36	0.12	0.56
Seg-8									-0.43	-0.17
Seg-9										0.12
Seg-10										

**Fig 8:**
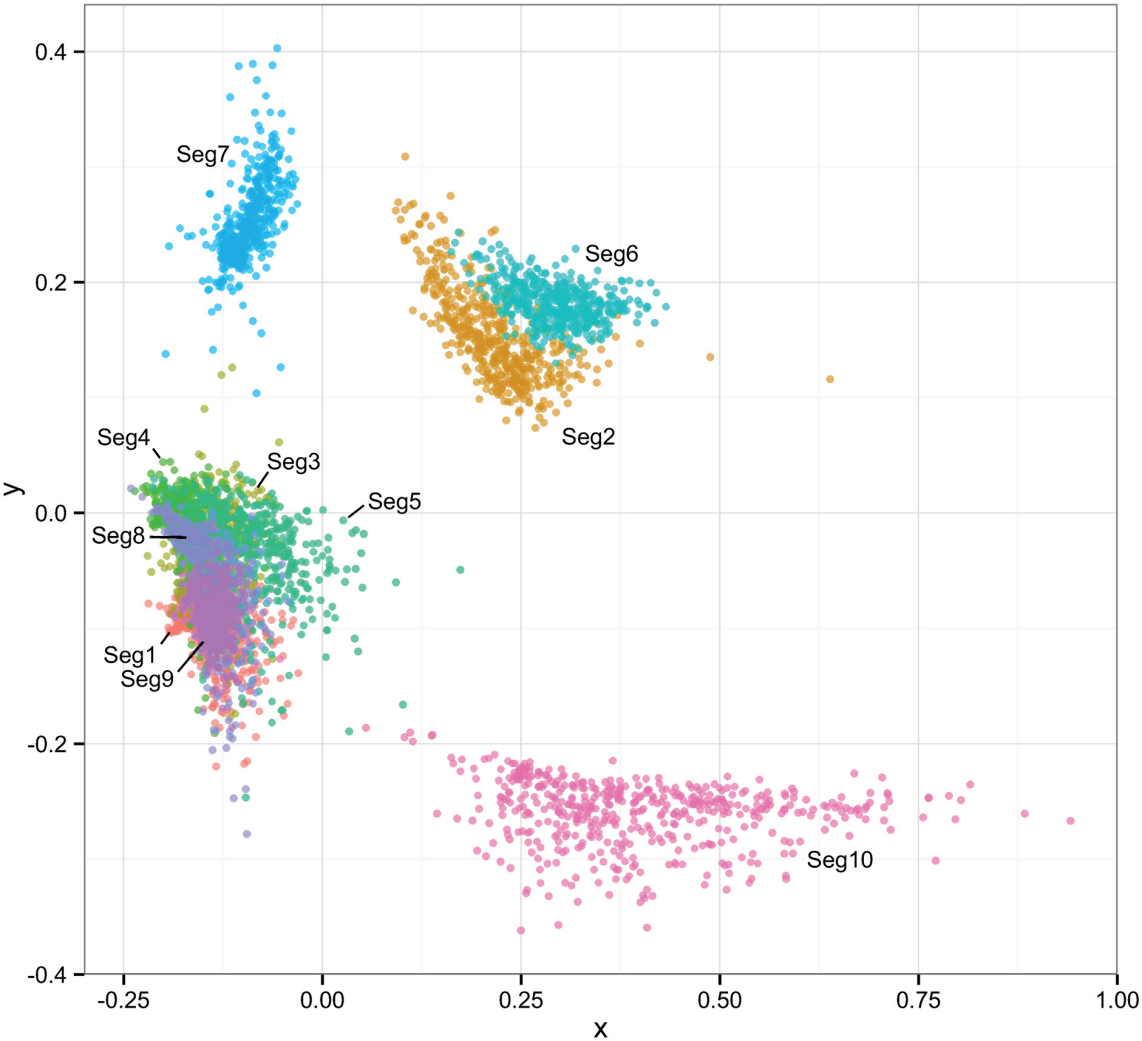
Multi-dimensional scaling plot reflecting correlations in the times to most recent common ancestor (tmrca) between pairs of European BTV viruses. Temporal estimation was done separately for each of the ten BTV segments to degree to which their evolutionary histories were shared. Clouds of points reflect phylogenetic uncertainty based on 500 trees sampled in program BEAST for each segment with pairwise comparisons to other segments being limited to viruses sampled in the same year. Only the first two dimensions of the scaling are shown in the plot.

The multi-dimensional scaling plot shows that the times to most recent common ancestor (tmrca) are broadly consistent between Seg-1, Seg-3, Seg-4, Seg-5, Seg-8, Seg-9 (Fig 8). By contrast, the tmrca of Seg-2, Seg-6, Seg-7 and Seg-10 do not overlap with any of the other segments, although Seg-2 and Seg-6 are in close proximity in the two-dimensional space. This suggests that *in vivo*, a core set of segments (Seg-1, Seg-3, Seg-4, Seg-5, Seg-8 and Seg-9) are less permissive to be broken up by reassortment than a second set comprised of Seg-2, Seg-6, Seg-7 and Seg-10.

### Associations between selection and reassortment

As found in previous studies [31, 32], purifying selection was by far the most dominant regime within the BTV genome with average dN/dS ratios ranging between 0.02 and 0.33 across the eleven open reading frames (Table 2). There was no relationship between the amount of selective constraint a segment experiences, as measured by its dN/dS ratio, and the frequency at which it reassorts (R^2^ = 0.074, p = 0.417). Small numbers of sites identified as positively selected by both the FEL and FUBAR methods were found in Seg-1, Seg-2, Seg-5, Seg-8, with the strongest signal of repeated adaptive change coming from Seg-9 (the gene encoding VP6 and NS4) where ten sites, all within VP6, were identified (Table 3). Although evidence for positive selection had been found for Seg-2, Seg-5 and Seg-9 before [32], the three codon sites previously implicated were different from the ones identified here. For Seg-9, we reconstructed changes in the ten positively selected residues along the phylogeny to determine whether non-synonymous changes took place more frequently at nodes where reassortment events had occurred. Out of the 169 nodes post-1955 assigned to European lineages in the Seg-9 tree, 8 had selection and reassortment, 15 had reassortment only, 13 had selection only and 133 had neither. According to these results, amino acid changes consistent with positive selection were about four times as common on nodes with reassortment than on nodes without (Fisher’s exact test, one-tailed, p = 0.002). Confirming the same pattern for the post-1993 data set was not possible, due to the number of nodes with reassortment being too small for meaningful analysis in this case. To test whether reassortants are selected against over time, we compared reassortment frequency at internal and tip nodes of the phylogeny but found a similar proportion of internal nodes with reassortment (7 out of 84) compared to tip nodes (11 out of 121, Fishers exact test, p = 0.530).

**Table 2:** Number of sites potentially under negative selection in the BTV genome according to two inference methods (FEL—fixed-effects likelihood, FUBAR—fast unconstrained Bayesian approximation). Mean dN/dS were estimated using the ‘single likelihood ancestor counting’ (SLAC) method. Number of reassortment events were estimated relative to Seg-2, which itself was assessed for reassortment relative to Seg-6.

		Codons under purifying selection			
	AA codons^1^	FEL	FUBAR	mean dN/dS	Reassortments
Seg-1	1302	1025	1212	0.045	11
Seg-2	992	926	978	0.160	1
Seg-3	901	783	878	0.020	8
Seg-4	644	471	582	0.066	4
Seg-5	552	410	513	0.065	4
Seg-6	529	513	523	0.060	1
Seg-7	349	317	344	0.023	3
Seg-8	354	244	282	0.109	7
Seg-9 NS4	79	40	49	0.095	4
Seg-9 VP6	330	23	11	0.333	4
Seg-10	229	151	182	0.052	7

^1^ Total number of codons in the alignment, with the actual number being variable among serotypes.

**Table 3:** Sites potentially under positive selection in the BTV genome according to two inference methods (FEL—fixed-effects likelihood [47], FUBAR—Fast Unconstrained Bayesian Approximation [48]). Sites with statistically strong evidence (p-value <0.05 and posterior probability >0.90) are shown in bold.

				FEL	FUBAR
Segment	Codon position	dS	dN	p-value	posterior probability
Seg-1	69	0	0.80	0.08	0.79
Seg-2	106	0	4.72	0.09	0.87
Seg-5	22	0	0.39	0.08	0.83
	211	0	0.67	0.05	0.87
Seg-8	244	0	1.98	0.03	0.86
Seg-9 NS4	78	0	6.96	0.19	0.91
Seg-9 VP6	5	0	3.78	0.06	0.95
	44	0	0.95	0.06	0.88
	47	0	0.97	0.07	0.87
	63	0	0.67	0.09	0.86
	66	0	1.08	**0.01**	**0.96**
	69	0	0.59	0.10	0.84
	72	0	2.17	**<0.01**	**0.99**
	75	0	1.38	**0.01**	**0.95**
	87	0	1.31	**0.01**	**0.96**
	93	0	0.45	0.09	0.85
	97	0	1.08	**0.03**	**0.92**
	98	0	0.63	0.07	0.88
	102	0	0.48	0.10	0.85
	103	0	0.94	**0.04**	**0.90**
	104	0	1.54	**0.03**	**0.92**
	111	0	0.83	0.06	0.89
	112	0	0.79	0.05	0.89
	114	0	0.97	**0.03**	**0.92**
	115	0	0.66	0.07	0.87
	117	0	0.63	0.07	0.88
	125	0	0.67	0.08	0.86
	126	0	1.04	**0.03**	**0.92**
	227	0	1.04	**0.04**	**0.91**

### Reassortment with modified live vaccines

To investigate whether live-attenuated vaccines have contributed to reassortment, we determined the distance between each vaccine strain and its closest genetic relative among the European field isolates. This produced repeated evidence for the reassortment and acquisition of segments from live vaccines (Table 4). For example, Seg-7 of the Spanish BTV-2 isolate [SPA2005/01], which was sampled at a time when live vaccines were being extensively used in a number of North African countries, is identical to that of a BTV-4 vaccine strain used in Southern Europe (0% uncorrected p-distance) [49]. Another three field strains were found to contain between two and six segments that closely matched known vaccine strains (Table 4).

**Table 4:** Evidence for BTV reassortment events involving modified live vaccines in Europe. For each segment, the distance from the vaccine strain (in substitutions) is shown. Also shown are the sampling location and year for the field strain and whether use of live-attenuated vaccine in Europe or surrounding countries had been documented for the relevant time period. Information on documented use of live vaccines was taken from Savini et al. 2008 [41].

Vaccine strain	Field strain	Location	Year	Nucleotide distance to vaccine strain										Documented live vaccine use?
				Seg-1	Seg-2	Seg-3	Seg-4	Seg-5	Seg-6	Seg-7	Seg-8	Seg-9	Seg-10	
9RSAvvv1-09	9ITL2003-01	ITA	2003		3	2			0					Bulgaria, Italy (1999–2006)
16RSAvvvv-16	16COR2004-01	COR	2004	1			1	0	1	0			0	Corsica (France), Italy (2004)
2RSAvvvv-02	2SPA2005-01	SPA	2005				0			1				Spain, Portugal (2000–2006), France (2000–2004), Italy (2002–2006)
BTV-4 RSA_vac	2SPA2005-01	SPA	2005							0				N Africa, Turkey, Spain, Italy (2004–2006), Portugal (2005–2006), Corsica (France, 2003–2004)

## Discussion

In this study, we provide a systematic assessment of BTV reassortment under field conditions that generates novel insights into the frequency, evolutionary constraints and consequences of this process. While previous studies have generated evidence of BTV reassortment, they were largely based on small, sub-genomic data sets, precluding specific insights into the frequency and patterns of association between segments. Our data confirm that genetic exchange by reassortment is a common and widespread phenomenon that, despite a time scale of only a few decades, has had a major impact on the genomic composition of European BTV strains. All lineages we sampled had undergone reassortment in their recent evolutionary past and in many cases this has taken place after viruses arrived in Europe. Given that our threshold for detection was chosen conservatively, the true extent of reasortment is likely to be even greater than documented here. While we don’t suggest that these findings are unique to Europe, our regionally focussed approach provided a particularly clear view of this process that would be hard to gain from studying areas of the world where BTV has been endemic for a long time.

As a result of reassortment, the genomes of BTV field strains are genetically highly variable, including substantial heterogeneity within the same serotype. The frequency with which reassortment takes place, appears to be largely driven by opportunity, suggesting a lack of any fundamental barriers keeping different BTV strains from exchanging genetic material during natural transmission. Indeed, our findings suggest that genetic ‘mixing’ is the norm whenever multiple strains co-circulate, mirroring observations from previous *in vitro* experiments [15].

However, natural reassortment does not appear to be a random process in BTV, consistent with findings from other segmented viruses [50–54]. While we found no significant bias in terms of individual segments or segment pairs being more likely to reassort than others, we detected a signal of non-random associations between segments during reassortment overall. This was also reflected in the MDS plot (Fig 8), depicting correlations in the time to most recent common ancestor between segments: Seg-7, Seg-10 failed to show any clear association with other segments and there was only weak evidence for a connection between Seg-2 and Seg-6. This suggests that these four segments experience few restrictions to being placed into different genomic backgrounds, implying protein functions that involve generalised interactions with proteins encoded by other genome segments. This finding is particularly noteworthy for Seg-2, which encodes the outer-capsid protein VP2 determining BTV serotype, since it suggests that serotype may be largely uncoupled from the phenotypic variation determined by other parts of the genome. Although the outer capsid proteins VP2 and VP5 (encoded by Seg-6) and the core surface protein VP7 (encoded by Seg-7) all interact extensively, it is thought that these interactions are not highly specific [55], consistent with the weak association found here.

In contrast, the remaining genome-segments (Seg-1, Seg-3, Seg-4, Seg-5, Seg-8, Seg-9) exhibit times to most recent common ancestor that are consistent among each other, suggesting physical, or biochemical interactions between their encoded proteins and consequently epistatic interactions that result in stronger evolutionary links. Some of these interactions are known: for example, VP1 (RNA-dependent RNA polymerase encoded by Seg-1), VP4 (capping enzyme including methyltransferase, encoded by Seg-4) and VP6 (RNA-dependent ATPase and helicase, encoded by Seg-9) form the viral replication complex and therefore it is reasonable that they need to coevolve in order to function optimally [56–59]. These viral enzymatic proteins are enclosed by layers of VP3 (subcore, encoded by Seg-3). The structural integrity of the core is essential for efficient transcriptional activity to be maintained [58,60]. In other cases however, the interactions suggested by our results are less well understood. Overall, these findings are broadly consistent with those reported recently for a smaller genomic data set from Australia, which also found Seg-7 and Seg-10 to evolve completely independently of the rest of the BTV genome, whereas all remaining segments showed at least some phylogenetic evidence of association with other segments. As in our work, for Seg-2 (VP2) this association was limited to Seg-6 (VP5).

The observed reassortment patterns indicate that under natural conditions, some genome segment combinations must be deleterious, causing these reassortants to be removed by purifying selection. This contrasts with findings from previous *in vitro* work, in which we were able to generate viable viruses representing all possible genome-segment combinations between two different BTV strains (BTV-1(w) and BTV-8(w)) by replacing segments one at a time [15]. Subsequent experiments with these reassortants further revealed no obvious phenotypic differences, neither with respect to *in vitro* growth kinetics nor for pathogenicity in an *in vivo* mouse model. However, more recent reverse genetics studies involving more distantly related BTV strains shows some combinations of reassortants cannot always be achieved at least using current methodology [61].

The notion that reassortment has fitness consequences for viruses circulating in the field is further supported by our findings regarding positive selection. We found evidence for adaptive evolution in a number of sites across the BTV genome, with the strongest evidence seen for ten sites in Seg-9, coding for VP6 and NS4. Following reassortment, amino acid changes were more common in these sites than would be expected by chance. This suggests that newly reassorted viruses are often under novel selective pressure, leading to adaptive genetic change that reflects the interplay of the proteins and/or RNAs derived from distinct parental origins. Our results point to physical or functional interactions between NS4, and/or VP6 and proteins encoded by other segments, consistent with the observation of Seg-9 maintaining close associations with most of the other segments (Fig 8). These findings also imply that a large proportion of reassortment changes must be detrimental, creating virus phenotypes with reduced fitness that are quickly removed from the population. They also indicate that genetic drift and shift are not entirely independent mechanisms but may be linked through functional constraints and changes in the individual RNAs and proteins that can either restrict or accommodate specific combinations of genome segments. Further experimental work, combining reverse genetics approaches with studies in animal models or arthropod vectors, will be needed to better understand this aspect of BTV biology.

We show that the live-attenuated BTV vaccine strains used in Europe over the past decades have repeatedly contributed segments to circulating ‘field’ strains, as suggested by earlier reports [38,43] and as seen on other continents [27]. Our data, representing a large set of full, European-wide genomes, demonstrates further cases of reassortment with live vaccines and shows that these events so far have involved all but two BTV segments (Seg-8 and Seg-9). The ability to detect evidence for reassortment between vaccine and field strains also implies that the frequency and transmission of progeny virus strains had risen to sufficiently high levels to be readily detected. This could reflect the widespread use of live-attenuated vaccines in certain periods and geographical locations, but could also indicate that the emerging reassortant strain accrues a fitness benefit relative to its parental strains and other reassortants from the same co-infection. Regardless of whether this is true, our results demonstrate that live BTV vaccines contribute to the genotypic and phenotypic variability of naturally circulating strains. This needs to be considered during the design and implementation of control strategies.

Our findings also have significant implications for BTV nomenclature and surveillance. Given the frequency at which reassortment occurs, Seg-2 (which determines virus serotype) can frequently become disassociated from the other genome segments within a specific virus lineage. Identifying BTV strains by serotype alone therefore reflects only 10% of the genome segments and does not reveal the potentially high level of genetic and phenotypic heterogeneity that exists within individual BTV serotypes. However, serotype remains an important indicator of strain relationships in antibody based neutralisation assays, and informs the choice of vaccine strain for control strategies and interventions. More generally, our work underscores the need for a nomenclature system for BTV and potentially other orbiviruses that reflects the entire genome rather than just one segment. With this in mind, using a system such as the one used in the orbivirus reference collection (www.reoviridae.org/dsRNA_virus_proteins/ReoID/BTV-isolates.htm) where virus isolates are identified individually by year, country of origin and isolate number, may be useful, so that differences between strains can be accurately recorded.

Comparing our inferred reassortment rate of around 0.05 per year to those of other segmented virus, such as influenza virus [51], is difficult because of methodological differences between studies. For example, we defined reassortment as segments changing their phylogenetic association from clade to another, with clades being at least 5% divergent. This would have ignored any events at finer genetic scales as illustrated by some of the field strains showing evidence of admixture with vaccine strains, which for some segments would not have been picked up by our conservative threshold. Regardless of the specific rate, reassortment is likely to be a major driver of genotypic and phenotypic change in BTV and might be more important in this respect than nucleotide substitutions. Comparative analyses of reassortment patterns and rates seen in different segmented RNA viruses, based on standardised approaches, would be useful and provide broader insights into the role of reassortment in virus evolution.

While our data revealed some of the general patterns, constraints and adaptive consequences of reassortment, many open questions remain. For example, although genetic exchange can take place in both vertebrate hosts and insect vectors [17,18], the relative contributions of these potential ‘mixing vessels’ to generating the observed patterns require further study. This includes importance of the individual insect as a genetic bottleneck fixing new variants (both point mutations and reassortants) within the virus population through founder effects and genetic drift [62,63]. We further hypothesise that the insect vector, which so far has been difficult to study experimentally, plays a critical role in determining the fitness of novel reassortant viruses. Similarly, it remains unclear to what extent reassortment might have facilitated faster invasion of novel host and vector communities in Europe, a question of much applied relevance that we are currently examining. Increasing our understanding of the biological mechanisms as well as the population-level consequences of BTV reassortment will be critical to improve our ability to prevent and control the global spread of this important livestock disease.

## Materials and Methods

### Virus isolates

Complete genomes were sequenced from 120 BTV virus isolates, from Europe and the Mediterranean region and African countries, collected during 1958–2012. These included 116 field isolates of BTV types 1, 2, 3, 4, 8, 9, 14, 15 and 16, as well as four live monovalent BTV vaccine strains of types 1, 2, 9, and 16 (S1 Table). Each of these virus isolates is included in the Orbivirus Reference Collection at The Pirbright Institute (www.reoviridae.org/dsRNA_virus_proteins/ReoID/BTV-isolates.htm) and is identified here by the corresponding reference number.

### RNA extraction, identification and typing of isolates

Total RNA was extracted from infected cell culture supernatants using the QIAamp Viral RNA Mini Kit (Qiagen) or Direct-zol RNA MiniPrep (Zymo Research) as per manufacturer's protocol. Identification and typing of BTV isolates was done by serogroup-specific real-time RT-PCR assays, targeting Seg-1 and Seg-10, and by specific conventional [64] and real-time RT-PCRs (available from Laboratoire Service International [LSI], Lissieu, France) targeting Seg-2. dsRNA was extracted from pellets of BTV infected cells using Trizol reagent (Invitrogen) as per manufacturer’s instructions for full-length cDNA synthesis. Viral RNA was analysed by agarose gel electrophoresis and used for whole genome sequencing.

While in principle some field isolates containing mixed infections could have resulted in reassortment during cell passage, this would have become evident during typing of the isolate (for Seg-2) or by the detection of mixed sequences (below), since strains were not plague-cloned and usually passaged only a few times. Mixed infections are therefore unlikely to have impacted our dataset.

### Genome sequencing

#### Sanger sequencing

Full-length cDNA copies of individual BTV genome segments were synthesised and amplified by reverse transcription-PCR (RT-PCR) using the ‘anchor-spacer-ligation’ method as described previously [65,66]. Individual cDNA amplicons were purified using the ‘GFX PCR DNA and gel band purification kit’ (Amersham Pharmacia Biotech, Inc) as per the manufacturer’s protocol. Sequencing of the quantified elutes was performed by Sanger sequencing using the BigDye terminator v3.1 kit (Applied Biosystems, Life Techologies,USA) on the 48-capillary 3730 DNA Genetic Analyzer (Applied Biosystems, Life Technologies, USA) according to the manufacturer’s protocols. Consensus sequences from each segment were assembled and analysed using DNASTAR Lasergene 11 (DNAStar Inc.).

#### 454 pyrosequencing

For each virus, equimolar, purified PCR products of the 10 genomic segments were pooled, and 1.5 to 2.5 μg of each sample was used for 454 pyrosequencing. All kits used for the preparation of the samples for sequencing were supplied by 454 Life Sciences, Roche, Branford, CT. Libraries were generated from the PCR samples using a GS FLX Titanium Rapid Library preparation kit, according to the manufacturer's instructions. Briefly, samples were nebulized with 30 lb/in^2^ of nitrogen for 1 min, followed by cleanup, fragment end repair, and adaptor ligation steps. GS FLX Titanium Rapid Library MID adaptors were used so that multiple libraries could be sequenced on the same region of a PicoTiterPlate device (PTP). After adaptor ligation, Agencourt AMPure beads (Beckman Coulter) were used for the removal of small fragments from the libraries. Amplification of libraries was carried out using a GS FLX Titanium emPCR kit and a pool of beads, consisting of 12 different MID-tagged libraries, was loaded onto one quarter of a PTP. Sequencing was performed on a GS FLX instrument (454 Life Sciences) according to the manufacturer's instructions.

BTV genomes were assembled against reference genomes of the same serotype by use of Newbler software (GS Reference Mapper, version 2.3; 454 Life Sciences). The assemblies were curated manually and edited using GAP4 [67] and for each virus, consensus sequences of each segment were generated as fasta files. For a few segments where incomplete consensus data were generated or sequencing ambiguities could not be resolved, complete finished genomes were obtained using capillary sequencing with BTV-specific primers.

The consensus sequences generated for individual genome segments were aligned with sequences for BTV genome segments from GenBank (S1 Table) (last access 31 August 2012) using MAFFT (http://www.ebi.ac.uk/Tools/msa/mafft/).

The data was initially checked for evidence of recombination using the program RDP as well as programs ‘MaxChi’, ‘GENECONV’, ‘BootScan’, ‘Chimaera’, ‘3Seq’ and ‘SiScan’ as incorporated in the RDP v.4.16 program [68]. No significant evidence was found for recombination within genome segments.

### Quantifying and dating reassortment events

The coding region of each genome-segment was aligned, according to the protein sequence, then converted to codon alignment using ‘PAL2NAL’ [69]. The 3’ and 5’ UTR regions were aligned using ‘Clustal Omega’ [70] and concatenated with the codon alignment for phylogenetic analysis.

The isolate’s age was estimated following Shapiro et al. 2011 [71] if one of the following applied: information about original sample date was missing (11 isolates); virus had been heavily passaged (>40 passages, 9 isolates); the number of passages was unknown (2 ‘old’ samples). Bayesian phylogenetic trees were estimated in ‘BEAST’ 1.7 [72] using a GTR+G+I model for the 5’ and 3’ UTRs and the SDR06 codon substitution model for the coding sequence [73]. Two Monte Carlo Markov chains were run for the number of generations needed for stationary distribution to be maintained after convergence (8 x 10^8^ generations sampled every 10000th generation). We used Tracer v1.6 [74] to visualize the posterior distribution for each parameter and obtain an estimate of the effective sample size (ESS). We assumed a run had converged if the ESS of all the parameters was above 100 when the two chains were combined. The trees from both chains were combined after removal of the initial 10% burn-in and resampled to provide approximately 10,000 trees. The maximum clade credibility (MCC) tree with mean node heights was produced from these trees using the auxiliary program TreeAnnotator, included in the BEAST package.

Cluster Picker [46] was used to define monophyletic clades on the MCC trees with posterior probabilities of 0.9 and a genetic distance threshold for clusters of 5%. The assignment to these clusters was subsequently used for character mapping of the clusters. Estimated phylogenies showed no evidence of clustering by host species.

The ancestral “cluster” state for each segment was summarized for each node of the maximum clade credibility tree over a distribution of Bayesian trees from Seg-2 using the parsimony reconstruction method in Mesquite [75]. In order to be accepted as a reassortment event, the majority of trees needed to contain that state as a uniquely best solution, according to the parsimony reconstruction. Because we are using conservatively defined clusters as our basis, our approach can only detect reassortment events involving sufficiently divergent lineages. In order to ensure that cluster switching was attributable to reassortment, as opposed to increased divergence due to continuous evolution, all reassortment events were confirmed visually on the phylogeny.

The date of each state change for each segment was subsequently extracted from the maximum clade credibility tree to determine the timing of reassortment events for nodes that had an ancestral node post-1900. The location of reassortment events was determined by reconstructing the character state (intra-Europe/extra-Europe) in the same way as above. Unless indicated otherwise, our analysis focussed on lineages that were assigned to Europe and that were sampled post-1998, as our sampling outside this spatial and temporal window was limited.

The number of reassortments per genome was calculated by dividing the total number of reassortment events in European lineages by the total branch lengths of European lineages scaled in time, using the BEAST consensus tree. To obtain an estimate of the number of reassortment events involving Seg-2, the ancestral state reconstruction was repeated for Seg-2 onto the maximum clade credibility tree for each of the other segments using a distribution of Bayesian trees. For illustrative purposes, we present results mapped onto Seg-6, as it had a small number of reassortment events itself. We used the ETE python tool to produce the phylogenetic figures [76].

To compare the segment phylogenies, multidimensional scaling plots were used to determine the tree-to-tree variation in branch lengths following the approach of Bahl [77]. Five hundred trees sampled from the MCMC chain for each segment were used to determine the time to most recent common ancestor (tmrca) for any pair of European taxa sampled within the same year (for years from 1998 to 2012). The correlation coefficient of tmrca estimates across all pairwise comparisons of trees was calculated and from it the tree-to-tree distance was estimated. The matrix of tree-to-tree distances was then plotted in two dimensions using multi-dimensional scaling.

We used simulations test whether the observed frequency at which segments were found to reassort differs from random expectations. Based on the observed number of reassortments, we draw samples of the same size from a vector of ten states with replacement and sorted the resulting sample by frequency. The mean and 95% range of frequencies encountered was calculated based on 10,000 replicates.

The checkerboard score was calculated from the matrix of genome-segment associations to test for the non-random co-segregation of individual segments. The C-score is compared to a null distribution of 1000 random matrices of the same size maintaining the number of reassortments per node but irrespective of the segment using the R packages ‘vegan’ [78] and ‘bipartite’ [79]. We further tested for significant positive and negative associations between segment pairs by determining Spearman’s rank correlation and corrected for multiple testing by controlling the false discovery rate.

To elucidate the potential role that vaccination strains may have played in reassortment, the distance from all European vaccine strains to their nearest sister clade among the European field strains was determined across all genome-segments. After initially using an uncorrected p-distance of 0.3% as a cut-off to classify a field isolate as ‘close’ to a vaccine strain, we determined in each case the number of substitution differences between field and vaccine strain.

### Adaptive responses to reassortment

To test whether increased selection occurs following reassortment, site-specific selection was estimated for all genes using the ‘fixed-effects likelihood’ (FEL) method [47] and the ‘fast unconstrained Bayesian approximation’ (FUBAR) method [48]. Sites under significant positive selection according to both methods (FEL: p-value <0.05; FUBAR: posterior probability >0.90) were mapped onto the appropriate gene tree, to determine the nodes where amino acid changes have occurred. We then tested whether reassortment had co-occurred on the same nodes more frequently than expected by chance. Mean dN/dS ratios for each segment were estimated using ‘Single Likelihood Ancestor Counting’ (SLAC) [47]. All selection analyses were performed in Datamonkey (http://www.datamonkey.org/).

### Nucleotide sequence accession numbers

All sequence data generated in this study have been deposited in GenBank (S1 Table, accession numbers KP820860—KP822064).

## Supporting Information

S1 Fig Number of BTV segments reassorting together per reassortment event. (EPS)

S2 Fig Time-scaled Seg-2 phylogeny of European BTV-1(e) isolates. No evidence of reassortment within Europe was detected within this data set. Shown is the maximum clade credibility tree estimated in program BEAST with branch lengths scaled in years. Inferred reassortment events (based on changes in genetic cluster assignment for one or more segments, shown on the right) are depicted next to tree nodes. Question marks in the genetic cluster assignments represented cases where the assignment was ambiguous (see Methods for further details).
(EPS)

S3 Fig Time-scaled Seg-2 phylogeny of European BTV-2(e) isolates. See S2 Fig for details.
(EPS)

S4 Fig Time-scaled Seg-2 phylogeny of European BTV-8 isolates. See S2 Fig for details.
(EPS)

S5 Fig Time-scaled Seg-2 phylogeny of European BTV-9(w) isolates. See S2 Fig for details.
(EPS)

S6 Fig Time-scaled Seg-2 phylogeny of European BTV-9(e) isolates. See S2 Fig for details.
(EPS)

S1 Table List of European BTV isolates included in this study and associated metadata, including accession numbers. (XLSX)

S2 Table Genome-wide evolutionary rate estimates for BTV. (XLS)
